# Tumor–Stroma Ratio in Colorectal Cancer—Comparison between Human Estimation and Automated Assessment

**DOI:** 10.3390/cancers15102675

**Published:** 2023-05-09

**Authors:** Daniel Firmbach, Michaela Benz, Petr Kuritcyn, Volker Bruns, Corinna Lang-Schwarz, Frederik A. Stuebs, Susanne Merkel, Leah-Sophie Leikauf, Anna-Lea Braunschweig, Angelika Oldenburger, Laura Gloßner, Niklas Abele, Christine Eck, Christian Matek, Arndt Hartmann, Carol I. Geppert

**Affiliations:** 1 Digital Health Systems Department, Fraunhofer-Institute for Integrated Circuits IIS, Am Wolfsmantel 33, 91058 Erlangen, Germany; 2 Institute of Pathology, University Hospital Erlangen, FAU Erlangen-Nuremberg, Krankenhausstr. 8–10, 91054 Erlangen, Germany; 3 Comprehensive Cancer Center Erlangen-EMN (CCC), University Hospital Erlangen, FAU Erlangen-Nuremberg, Östliche Stadtmauerstr. 30, 91054 Erlangen, Germany; 4 Institute of Pathology, Hospital Bayreuth, Preuschwitzer Str. 101, 95445 Bayreuth, Germany; 5 Department of Obstetrics and Gynaecology, University Hospital Erlangen, FAU Erlangen-Nuremberg, Universitätsstraße 21–23, 91054 Erlangen, Germany; 6 Department of Surgery, University Hospital Erlangen, FAU Erlangen-Nuremberg, Krankenhausstr. 12, 91054 Erlangen, Germany

**Keywords:** segmentation, deep learning, image analysis, few-shot learning, U-Net, tumor–stroma ratio, colorectal cancer

## Abstract

**Simple Summary:**

A lower tumor–stroma ratio within a tumor correlates with a poorer outcome, i.e., with a higher risk of death. The assessment of this ratio by humans is prone to errors, and when presented the same case, the ratios reported by multiple pathologists will oftentimes deviate significantly. The aim of our work was to predict the tumor–stroma ratio automatically using deep neural segmentation networks. The assessment comprises two steps: recognizing the different tissue types and estimating their ratio. We compared both steps individually to human observers and showed that (i) the outlined automatic method yields good segmentation results and (ii) that human estimations are consistently higher than the automated estimation and deviate significantly for a hand-annotated ground truth. We showed that including an additional evaluation step for our segmentation results and relating the segmentation quality to deviations in tumor–stroma assessment provides helpful insights.

**Abstract:**

The tumor–stroma ratio (TSR) has been repeatedly shown to be a prognostic factor for survival prediction of different cancer types. However, an objective and reliable determination of the tumor–stroma ratio remains challenging. We present an easily adaptable deep learning model for accurately segmenting tumor regions in hematoxylin and eosin (H&E)-stained whole slide images (WSIs) of colon cancer patients into five distinct classes (tumor, stroma, necrosis, mucus, and background). The tumor–stroma ratio can be determined in the presence of necrotic or mucinous areas. We employ a few-shot model, eventually aiming for the easy adaptability of our approach to related segmentation tasks or other primaries, and compare the results to a well-established state-of-the art approach (U-Net). Both models achieve similar results with an overall accuracy of 86.5% and 86.7%, respectively, indicating that the adaptability does not lead to a significant decrease in accuracy. Moreover, we comprehensively compare with TSR estimates of human observers and examine in detail discrepancies and inter-rater reliability. Adding a second survey for segmentation quality on top of a first survey for TSR estimation, we found that TSR estimations of human observers are not as reliable a ground truth as previously thought.

## 1. Introduction

The tumor–stroma ratio (TSR) has repeatedly been shown to be a prognostic factor for the survival prediction of patients suffering from a number of malignant diseases, including colorectal [1,2,3,4,5,6,7,8,9,10,11,12,13,14], lung [15,16,17], liver [18], or breast cancer [19,20,21,22,23]. The most common scoring method, especially for colon cancer, was developed by van Pelt et al. [7], although the individual methods can vary.

However, only a few of these works focus on computer-assisted estimation, although they hold the promise of providing a more objective and a less error-prone method, especially when using machine-learning models.

Early computer-assisted approaches for tumor and stroma assessment were based on conventional machine learning, using so-called handcrafted texture features. For example, Linder et al. [24] investigated various texture features such as local binary patterns and Gabor filters together with support vector machines (SVM) for the classification of image patches as either tumor epithelium or stroma. Image patches were extracted from digitized colorectal cancer tissue microarrays (TMAs) which had been immunostained with an epidermal growth factor receptor antibody. TSR values were not calculated but rather mentioned as a possible application. Similarly, Bianconi et al. [25] applied handcrafted features in conjunction with an SVM as well as a nearest-neighbor and naive Bayes rule classifier to address the same two-class problem (tumor epithelium vs. stroma) on colorectal cancer TMAs, but they did not calculate TSR values either. Geessink et al. (2015) [26] investigated the pixel-wise classification of tumor and stroma tissue in hematoxylin and eosin (H&E)-stained digitized colon sections. They also used explicit features, for example local density nucleus pixels, and trained a normal-density-based quadratic classifier. They evaluated their approach against manual pixel-wise annotations. Since they only distinguished between tumor and stroma, smaller necrotic areas were counted as part of the tumor. In subsequent work, Geessink et al. (2019) [8] applied a convolutional neural network (CNN) for tissue classification and distinguished nine different tissue classes. The computer-derived TSR values were compared to TSR values estimated by two pathologists for 129 patients, which was followed by a survival analysis. Zhao et al. [9] also applied a CNN that was trained on image patches of nine different tissue classes. In contrast to many other approaches, which calculate the TSR only within a region of interest (ROI), they determined the TSR based on the complete WSI as a ratio between the classified stroma and tumor areas. In addition to evaluating the prognostic significance of the TSR regarding the survival rate, they also performed a TSR consistency analysis on 126 images with manual annotations of stroma and tumor tissues. However, for this consistency analysis, they hand-selected ROIs that only comprised tumor and stroma tissues. Millar et al. [22] used the QuPath PixelClassifier v0.2.1, yet they only segmented the images into the three classes: tumor epithelium, stroma and background (with fatty tissue). They calculated the TSR for breast cancer TMAs that were stained with H&E and investigated the prognostic significance of the TSR. Segmentation results were not quantitatively evaluated, but the authors indicated that segmentation required supervision by a pathologist and reported this as a limitation of their study. They speculated that a deep learning approach might improve segmentation. Hacking et al. [27] used QuPath superpixel image segmentation (SIS) together with an artificial neural network as a classifier to segment tumor regions into tumor epithelium, collagenous stroma and myxoid stroma. They also did not quantitatively evaluate their segmentation results but focused on the prognostic value of myxoid stroma ratio. Hong et al. [28] only considered three classes: background (non-tissue), stroma and tumor. They generated a binary tissue mask with a fixed threshold after transformation of the H&E image into grayscale. The special aspect of their work is converting the grayscale H&E image into a virtual cytokeratin stained image using a conditional generative adversarial network (GAN). Afterwards, they binarized the cytokeratin image by thresholding its chromogen (Diaminobenzidine, DAB) channel. Based on these two binary masks, they calculated the tumor and stroma areas. Abbet et al. [12] reported a fully automated TSR estimation on WSIs and performed survival analysis on 221 WSIs of colorectal cancer patients. Their TSR scoring followed the recommendation of Pelt et al. [7]. In a first step, a tissue classification is performed on the full WSI by applying a model trained using self-supervision and unsupervised domain adaptation [29] to detect tumor and tumor-adjacent stroma tissue. As in the other CNN-based classification methods mentioned above, a class label is assigned to every image patch. The resulting checkerboard-like segmentation is then smoothened by applying conditional random fields. In a second step, automatic identification of the ROI in which the TSR is to be determined takes place. Finally, TSR is calculated within this ROI. Moreover, the TSR for the complete WSI is calculated, and both TSR values were shown to be statistically relevant for survival analysis. Smit et al. (2023) [14] investigated how feasible semi-automated and fully automated TSR scoring is. They used the same procedure as Geessink et al. (2019) for tissue segmentation. For the semi-automated approach, the ROIs from the visual scoring were used. For their fully automated approach, the WSIs were segmented and post-processed by a concave hull algorithm to obtain the tumor region. They selected circular ROIs based on several rules such as, e.g., size or lack of background.

Most of the works cited above focus on survival analysis based on TSR scoring [8,9,12,22,28]. An evaluation of the TSR values compared to those of human observers was performed by Geessink et al., Hong et al. and Smit et al. [8,14,28]. Geessink et al. performed both a comparison of the TSR values and a pixel-by-pixel comparison of the segmentation results against manual annotations. Zaoh et al. [9] performed a thorough evaluation of the segmentation and TSR determination of their method, but they only considered the tumor and stroma.

Our work combines the most important aspects of previous works. We segment a wide number of relevant tissue types in the tumor microenvironment (TME), including the tumor, stroma, necrosis, mucus and background. Therefore, with our approach, the TSR can even be determined in regions where necrosis or mucus are present. Almost all of the methods mentioned above are based on patch-wise classification. We directly employ a more finely grained segmentation method. The main difference between segmentation and classification approaches is that the result of the former is a mask that assigns the contained pixels of the considered image patches to the different classes, while classification assigns the whole image patch to a single class. Classification yields a detailed segmentation map by analyzing overlapping image patches (increasing the computational complexity) and by post-processing, e.g., using conditional random fields.

The supervised training of a deep learning-based segmentation approach requires pixel-precise annotations of a set of example images. Creating such annotations is a very time-consuming and tedious task. Therefore, we have chosen a so-called few-shot method [30] instead, which can be adapted to new segmentation tasks given only a few new annotated examples (“a few shots”). In the case of prototype-based few-shot models, this strategy can even be used without retraining the underlying neural network’s weights. In contrast, only prototypes representing the classes to be segmented need to be adjusted, which may represent one of the most attractive features of this few-shot approach. As a comparative baseline reference, we train and evaluate a U-Net model [31], which is one of the most widely applied algorithms for the segmentation of biological and medical image data [32]. We present both an evaluation on a pixel-wise annotated test set and a comparison of human observers’ TSR values with the TSR values derived from the predicted segmentation results. Moreover, we examine in detail the causes of discrepancies between the calculated and estimated TSR values as well as the inter-rater agreement.

## 2. Materials and Methods

### 2.1. Data

All the image data analyzed in this work originated from H&E-stained colon tissue sections containing adenocarcinoma of various grades. They were digitized using a 3DHistech MIDI scanner at a resolution of 0.22 μm per pixel at the University Hospital Erlangen, resulting in whole slide images (WSIs). All WSIs were generated in a retrospective study approved by the scientific committee (CCC tissue biobank) of the Comprehensive Cancer Center (CCC Erlangen-EMN; application-No. 100030; date of approval 09.05.2012) of the Friedrich-Alexander University Erlangen-Nuremberg. The study was based on the approval of the Ethics Commission of the University Hospital Erlangen (No. 4607 from 18.01.2012). The study was performed in accordance with the Declaration of Helsinki, and ethical guidelines relevant for retrospective studies were respected throughout. Tissue histology was reviewed by two board-certified pathologists (AH and CG). Pathology reports and medical records of patients who underwent an operation at our hospital were reviewed.

We selected 59 patients diagnosed with colon cancer at University Hospital Erlangen, Germany between 1999 and 2006. All patients received primary surgical resection without neo-adjuvant therapy. The median age at date of diagnosis was 67 years with a minimum of 31 years and a maximum of 90 years. Overall, 37 individuals were male and 22 were female, reflecting a typical distribution of gender and age in colon cancer.

Tumor grade was 2 in 37 patients and 3 in 22 patients. No grade 1 tumor was included. The distribution of tumor stage (TNM 2017) was pT1 (n = 4), pT2 (n = 12), pT3 (n = 40) and pT4 (n = 3), respectively. The lymph node status was negative in 35 (pN0) and positive in 24 (pN+) cases. The tumor size was median 45–40 mm (min 10 mm and max. 110 mm, respectively). All individuals had invasive adenocarcinoma of the colon; there was no other primary or secondary entity. The carcinoma tissue was analyzed without taking adenoma tissue into account if present. With the exception of diverticulosis, no further relevant finding was reported by pathologists.

All patients received state-of-the-art open surgery with complete resection (R0) (extended mesocolon resection (no laparoscopic surgery)) including representative amount of lymph nodes harvested (median: 34 lymph nodes; minimum 10 and maximum 97).

No other malignoma or metastases of primaries other than the colon were known. Only 1 of 59 patients suffered from metastatic disease, which was in this case to the liver (pM1(HEP)). Subtypes were represented as follows: most of the cases were tubular or cribriform typical adenocarcinoma non-special other type (NOS) (n = 46), followed by mucinous type (n = 8), medullary type (n = 4) and signet ring cell carcinoma (n = 1). This reflects a typical distribution of subtypes in colon cancer.

#### 2.1.1. Segmentation Dataset with Pixel-Wise Annotations

A new dataset with pixel-wise annotations was established, where each pixel was assigned to one of the six classes: tumor, stroma, necrosis, mucus, background and artifact. First, 44 regions of interest (ROIs) were selected manually from a set of 33 WSIs. These regions had an average size of 1 mm × 1 mm, which corresponds to roughly 4500×4500 pixels in the native scan resolution. The WSI represented a tumor slide of routine work with the tumor center and invasion front as well as the relevant growth pattern and grade of each case. The location of the ROIs was chosen such that all the tissue classes of interest (tumor, stroma, necrosis, mucus) as well as different tumor grades were present, if possible.

Afterwards, all ROIs were annotated, and the annotations were revised and approved by an experienced senior pathologist (CG). Finally, the dataset was split into three disjoint sets: one for training of the neural segmentation networks (training set), one for model selection (validation set) and one for evaluation of the segmentation models (test set). The training set contained 29 ROIs from 23 WSIs, the validation set contained 6 ROIs from 4 WSIs, and the test set contained 9 ROIs from 6 WSIs. Table 1 provides an overview of the three datasets.

#### 2.1.2. Survey Dataset

A second dataset was created to compare the TSR values of both models to TSR values estimated by human observers. Therefore, care was taken to reflect the procedure in routine clinical practice for TSR determination: first, a representative ROI of a defined size within the tumor has to be selected. Then, the TSR is estimated by eyeballing without performing any manual segmentation within the image. The dataset is comprised of 30 ROIs sized 2 mm × 2 mm, each from a different WSI. All ROI positions were determined by an experienced pathologist (CG), ensuring that all observers estimate the TSR based on the same ROI. The ROIs are stored in the original resolution (0.22 µm/pixel).

No image sections from any of these WSIs were part of the training or validation set of the “Segmentation Dataset”. Additionally, in two of these ROIs, the tumor and stroma tissue were manually annotated to allow a more in-depth comparison between the calculated and estimated TSR values for these two examples.

### 2.2. Segmentation Methods

All of the following methods were implemented using the TensorFlow framework (version 2.3.0) [33].

**Figure 1:**
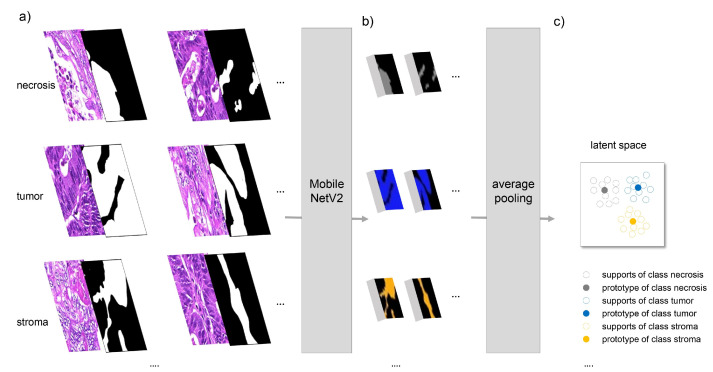
In the BPN approach, each segmentation class is represented by so-called prototypes in latent space. Prototypes are calculated based on a few support examples per class. (**a**) For each class, pairs of image patches (512×512 pixels) and corresponding annotation masks are given. Image patches are propagated through an adapted MobileNetV2, resulting in a downsampled feature mask of size 32×32 pixels. (**b**) Feature masks are fused with the downsampled annotation masks. (**c**) Subsequently, average pooling with respect to the annotation mask is applied, resulting in one feature vector for each support in latent space. The prototype for each class is given by the mean over all supports.

Two different deep learning-based segmentation methods were applied for the segmentation of the image pixels into the five classes (tumor, stroma, necrosis, mucus, and background). The first one was a so-called prototype-based few-shot approach. The main idea is that assignment to a class is made based on the similarity to class prototypes in a feature space (also called latent space) learned by the neural segmentation network. Specifically, we used a reduced version of PANet, which was introduced by Wang et al. [30]. We did not use prototype alignment regularization, which led to a simpler model we denote the Basic Prototype Network (BPN). We furthermore used a modified MobileNetV2 [34] as a feature extractor instead of the VGG-16 used in the original PANet due to its significantly lower number of parameters and memory requirements. We removed all layers of MobileNetV2 after the 16th bottleneck layer as well as the last convolutional layer of the 16th bottleneck layer, resulting in appropriately sized feature masks. Retaining all layers would decrease the height and width of the feature mask by a factor of 32 each relative to the size of the input image. Through this modification, the input size was only reduced by a factor of 16. We also tested other variants of MobileNetV2 with an even bigger size of the feature mask, but we obtained worse results on the segmentation dataset with that approach. As a similarity measure, we used the negative squared Euclidean distance with a prefactor of 0.5 instead of the cosine distance. Figure 1 visualizes the determination of class prototypes. For each class, some sample images (“supports”), including pixel-precise annotation of this class, were required. In our setup, these images had a size of 512×512 pixels. The images were propagated through the modified MobileNetV2, resulting in a downsampled feature mask of size 32×32 pixels. Using the similar downsampled annotation mask, masked average pooling was applied to the corresponding pixels in the feature mask. As a result, one feature vector representing the average of all pixels belonging to this class in this support image was obtained. Finally, the class prototype was calculated by averaging all supports of this class. “Query” images to be segmented were also propagated through the network, resulting in feature masks of size 32×32 pixels. For all pixels in these feature masks, the similarity to all class prototypes was calculated, and each pixel was assigned to the class of its closest prototype. Lastly, the segmentation results were bilinearly upsampled in order to obtain a mask in the full native resolution.

The second segmentation method used a U-Net structure [31] with the typical contracting and expansive paths interconnected with horizontal connections. The input for the contracting path included images of size 512×512 pixels. The path used exactly the same modified MobileNetV2 as described for the BPN case, extracting feature masks of size 32×32 pixels. These were then used as the starting point for the expansive path. This path consisted of four blocks. Each block started with an upsampling layer, whose output was concatenated with the output of the last bottleneck layer of the contracting path with the same spatial size. Afterwards, two convolutional layers with batch normalization and a ReLU activation layer followed. The number of filters in the convolutional layers is provided in Appendix A. At the end of the fourth block, the final convolutional layer without batch normalization nor an activation layer reduced the number of channels to the number of classes.

### 2.3. Training

The BPN models were trained with episodic training for up to 10,000 episodes. The input to each episode was five supports per class and two query images with their corresponding annotation masks. We used the Adam optimizer and a learning rate of $5\times 10^{-4}$. The α parameter of the modified MobileNetV2 was set to 1.0. As the loss function, a pixel-wise adaptation of the COREL-loss [35] with a γ value of 0.5 was chosen. The U-Net models were trained for up to 5000 steps with a batch size of 25, 5 images per class, using the Adam optimizer and a learning rate of $2\times 10^{-4}$. The α parameter of the modified MobileNetV2 was set to 0.75. A lower value of α was used to keep the number of parameters between BPN and U-Net comparable. As the loss function, a pixel-wise cross-entropy was used. For both models’ initialization, weights that were pre-trained on the ImageNet dataset [36] were used. Data augmentation was performed, following Kuritcyn et al.’s [37] suggestion of H&E color, hue, and saturation augmentation. Afterwards, they were transformed to have zero means and a standard deviation of 1.

The models were validated every 1000 steps (U-Net) or episodes (BPN), and the weights with the best validation score were saved. Both BPN and U-Net were trained three times with identical hyperparameters.

### 2.4. Evaluation Protocols

Both tasks—tissue segmentation into five classes and TSR estimation—were evaluated separately. Thus, we used multiple evaluation protocols. The first is a pixel-wise evaluation of our segmentation approaches on the test set of the “Segmentation dataset” (see Section 2.1.1). This set was manually annotated, and a quantitative comparison to the ground truth annotation was performed. The evaluation details are described in Section 2.4.1.

The evaluation of the TSR estimation comprised three components. Similar to other work [8,28], the calculated TSR values were compared with TSR values from human observers. Our evaluation included less WSIs than the, e.g., Geessink et al. (2019) [8] or Hong et al. [28], but the TSR estimates were provided by more observers with different experience levels. Details on this evaluation are provided in Section 2.4.2. In addition, a second evaluation assessed the mean segmentation quality of the models and whether there was a direct relationship between the mean segmentation quality and the deviation in TSR values between AI model and human observers (see Section 2.4.3). Finally, for two selected ROIs, a time-consuming pixel-wise annotation was created and used as the gold standard for TSR estimation.

#### 2.4.1. Pixel-Wise Evaluation of Segmentation Approaches

The pixel-wise evaluation was performed on the segmentation test set. Since the test images were larger than the image patches processed by the segmentation models, tiling of the test images was required. Two different tiling methods were used. The first one (denoted as tiling A in the following) was performed without overlap. Only at the right and lower boundary did an overlap occur, because the width and height of the test images were not multiples of the edge lengths of the image patches. Within the small overlapping area, no averaging was performed, but the segmentation results of the last added patch were taken. The second tiling method (denoted as tiling B in the following) discarded the segmentation results for all pixels within the outer boundary of each patch except for the boundaries of patches that coincide with the test image border, since the test image would not be complete otherwise. The width of the boundary was set to 96 pixels for all experiments, reducing the active output area per patch to 320×320 pixels (39%). To fully segment the test image, input patches therefore had to overlap, whereby the overlap was twice as wide as the boundary. Finally, the segmentation results of all test images were compared to their manual ground truth annotation by pixel, resulting in a confusion matrix. Based on this confusion matrix, other metrics such as recall, precision, intersection over union (IoU), and F${}_{1}$ score were calculated (see Appendix C for further details).

Results were obtained by evaluating all three training runs and averaging the results for each combination of segmentation and tiling methods. In case of the BPN approach, in addition to the three training runs, the support set was also altered. A total of $N_{S}=100$ supports were randomly drawn from the training set and prototypes were calculated. Afterwards, these prototypes were used for evaluation on the test set together with the underlying model. Every model was evaluated ten times with different supports, resulting in 30 evaluations, and the results were averaged.

#### 2.4.2. Comparison between Human Estimation and Automated TSR Assessment

**Figure A1:**
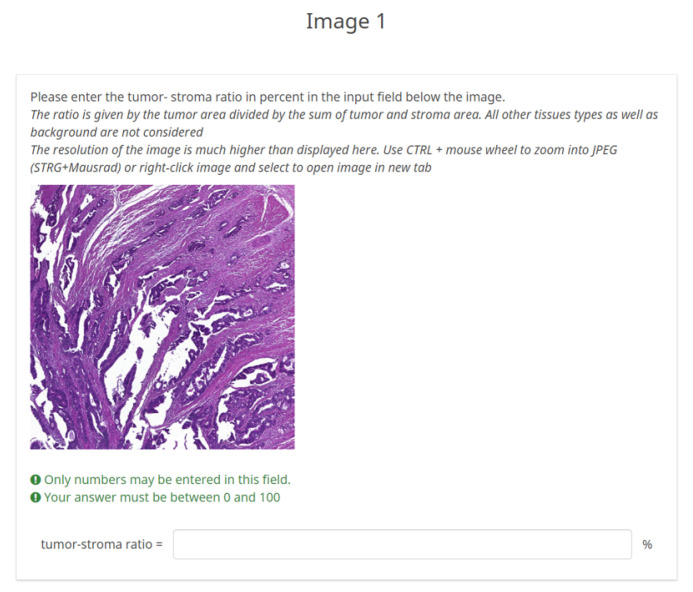
Example view of the online survey.

An online survey (survey 1) was conducted in which a total of 10 observers, including pathologists of different levels of experience and trained medical students, were asked to estimate the TSR for each ROI of the survey dataset (Figure A1). For all ROIs, the TSR was also calculated based on the segmentation results of both models (BPN and U-Net). Hereby, the tumor and stroma areas were given by the total number of pixels predicted as tumor and stroma, respectively. Pixels assigned to the classes mucus, necrosis or background were not counted toward the tumor or stroma area. The TSR was calculated as the ratio between the tumor area and the sum of tumor and stroma areas. In the following, these values will be referred to as AI-TSR values. Inter-observer agreement was assessed using a two-way mixed effects, single-measurement, absolute agreement intraclass coefficient (ICC). In addition to the overall ICC, ICCs of different observer groups, based on their experience level, were determined as well. These groups were: senior (three observers), junior (three observers), and beginner (four observers). For further analysis, only the results of the two most experienced observers (m.e. observers) were used. Furthermore, the ICC and Cohen’s kappa (using categorized TSR) for the m.e. observers were calculated. Agreement in TSR estimation was assessed using the ICC and Cohen’s kappa between the m.e. observers and AI models. Cohen’s kappa was calculated using categorized TSR. The categories used were stroma-low (>50% TSR) and stroma-high (<50% TSR). Additionally, a threshold of 65% for stroma-low to stroma-high separation was used, following Hong et al. [28] and Geessink et al. (2019) [8].

#### 2.4.3. Survey 2—Assessment of Segmentation Quality

An additional survey (survey 2) was conducted to assess the quality of segmentation results obtained with the BPN model for all 30 ROIs. One senior pathologist and a trained medical student independently graded the segmentation quality on a scale from 1 (insufficient segmentation) to 10 (excellent segmentation). The mean of their grades was used for final quality assessment. This assessment was used to further examine the difference between human and automated TSR estimations. The assumption was that a high rating of segmentation quality with large TSR discrepancies between human and AI models indicates human error or bias, whereas a poor rating of the segmentation quality indicates errors of the automated TSR assessment.

## 3. Results and Discussion

### 3.1. Pixel-Wise Segmentation

**Figure 2:**
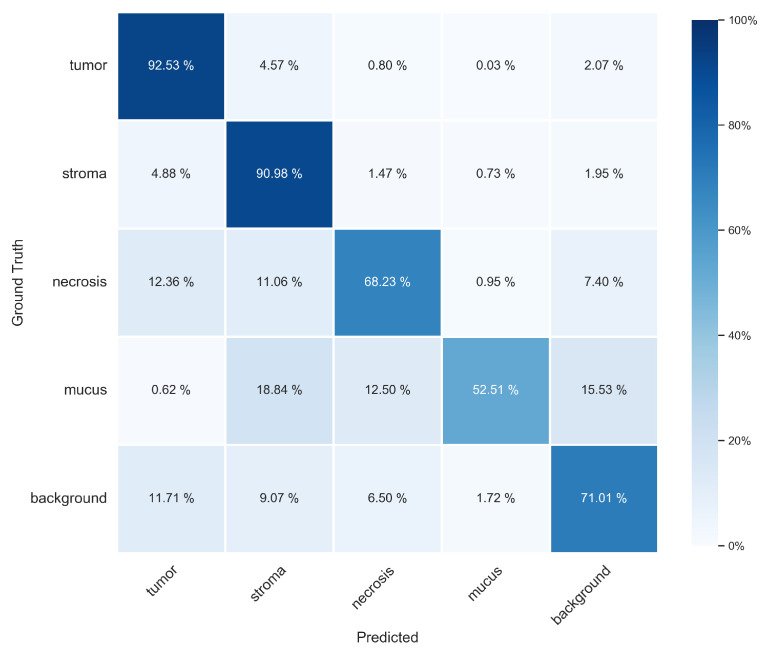
Confusion matrix obtained with the BPN model on the segmentation test set applying tiling B. The rows represent the ground truth class labels, and the columns represent the predictions. Thereby, the rows of the matrix are each normalized to 100% based on the number of pixels of each class in the test set.

Results of the pixel-wise evaluation on the segmentation test set for both methods (BPN and U-Net) with both tiling methods are given in Table 2. Applying tiling B yielded better results than tiling A. This might be expected, since poorer segmentation results of pixels near the boundary of each patch were discarded in tiling B. For these pixels, less context information is available from the neighborhood due to their position near the boundary, which leads to less accurate results. The higher overall accuracies compared to the means of the other metrics are due to the imbalance of the class distribution. The two classes of tumor and stroma were more frequently represented than the others (Table 1), and the segmentation of these classes was performed much better than that of the under-represented classes of mucus, necrosis and background. This is reflected in higher F${}_{1}$ scores (Table 3) as well as in the confusion matrix (Figure 2). Class-specific recall, precision and IoU values (Table A2, Table A3 and Table A4) show a similar trend as the F${}_{1}$ scores.

A closer inspection of the confusion matrix (Figure 2) and the class-specific metrics such as F${}_{1}$ score (Table 3) indicates that the worst performance was achieved for necrosis and mucus. Mucus was frequently misclassified as stroma, background or necrosis, whereas necrosis was often misclassified as tumor, stroma and background. The pixel-precise distinction between background, mucus and necrosis was equally challenging during the ground truth annotation in areas where these three classes were mixed together, since transitions between these classes are vaguer than those between the other classes.

**Figure 3:**
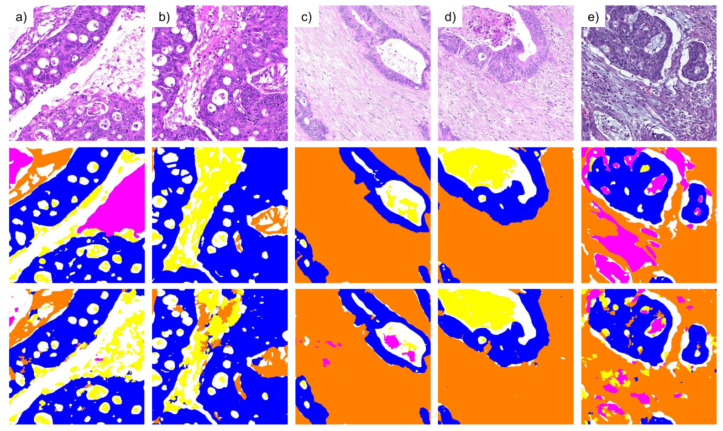
The first row shows original image regions, the second row shows the corresponding hand-annotated ground truth, and the third row shows the segmentation results obtained with the BPN approach and tiling B. Each image region has an approximate size of 440 µm × 440 µm. Columns (**a**–**e**) show different image regions. Class labels are color-coded: blue (tumor), orange (stroma), yellow (necrosis), magenta (mucus), white (background).

Examples of segmentation results obtained with the BPN approach and tiling B are shown in Figure 3. The difficulty of separating background, necrosis and mucus is apparent in the images of columns (a) and (b). Columns (c) and (d) demonstrate the ability for high-quality segmentation results of both stroma and tumor with only minor missegmentation. Column (e) shows an example of mucus misclassified as stroma.

Comparing the BPN and U-Net approaches shows a slightly better performance of the latter on the segmentation test set (Table 2). However, the advantage of using a few-shot based method lies in its adaptability to new tasks or data distributions with no need for additional training. We conducted a preliminary experiment, where we compared a BPN to a fine-tuned U-Net in the task of gland segmentation, with only a handful of image patches for either prototype calculation or fine-tuning. The BPN did not only perform better, but its adaption consumed less computation time, since no model re-training was involved. It only required calculation of new prototypes based on a few example annotations (Figure 1), which can be completed in a couple of seconds.

### 3.2. Evaluation of TSR Assessment

**Figure 4:**
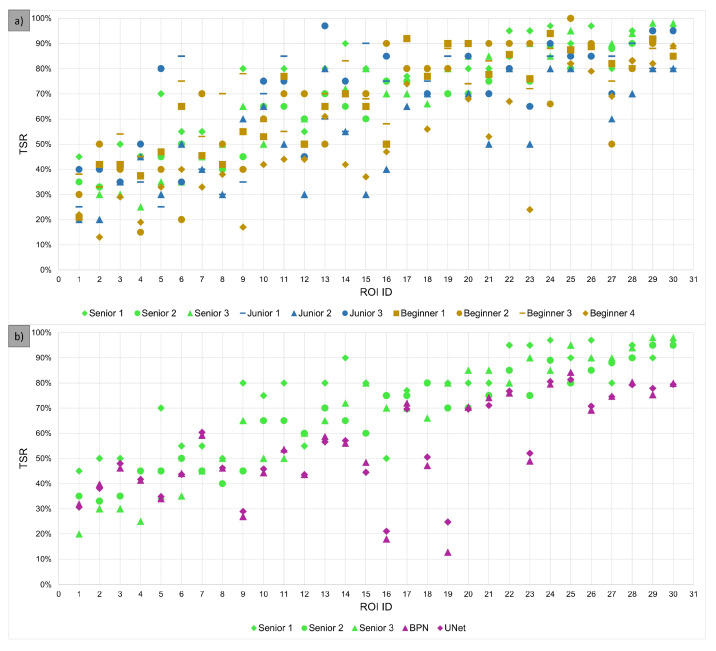
TSR estimations (y-axis) for each of the 30 ROIs from (**a**) human observers as well as (**b**) senior observers, BPN and U-Net method. The ROI ID (x-axis) was assigned based on the average TSR estimation of senior 2 and senior 3 (m.e. observers), which means the ROI with ID 1 has the lowest TSR value in the m.e. observers estimates, and the ROI with ID 30 has the highest value. Individual observers and both models are represented by different colors and shapes. A color represent either one of three observer groups or the models. Shapes are used to differentiate between members of single groups.

Figure 4 shows all TSR estimations from all observers (Figure 4a)) as well as the AI-TSR values obtained with the BPN and U-Net approach, together with senior observer values (Figure 4b)) for all 30 ROIs of the survey dataset. Individual estimations for one particular ROI can differ significantly between observers. Even the ROIs with the closest agreement of estimates varied up to roughly 20 percentage points. Both the BPN and U-Net approaches yielded almost identical TSR values. For ROIs with higher TSR values (starting at about 50%), they tended to estimate a lower TSR value compared to human observers.

The ICC between all observers was 0.673 (95% CI 0.54–0.80), showing moderate to good agreement according to Koo et al. [38]. The beginner and junior group had ICC values of 0.617 (95% CI 0.40–0.78) and 0.594 (95% CI 0.36–0.77), respectively, both showing poor to good agreement. The senior group had an ICC of 0.788 (95% CI 0.61–0.89), showing moderate to good agreement. The two most experienced observers (senior 2 and senior 3)—denoted as m.e. observers in the following—had an ICC of 0.87 (95% 0.75–0.94), showing good to excellent agreement. Cohen’s kappa between them was 0.734, showing a substantial agreement according to Landis and Koch [39].

This value is consistent with previously stated values: 0.578 for Geessink et al. (2019) [8]; 0.68 for Roeke et al. [21]; 0.74 for Moorman et al. [19]; 0.83 for Smit et al. [11]; and 0.89 for Huijbers et al. [3]

**Figure 5:**
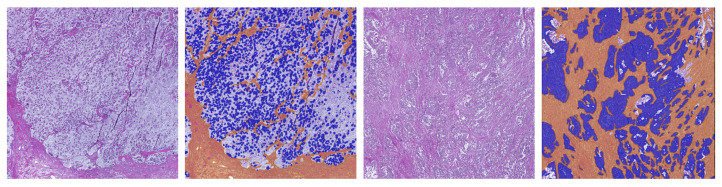
Manual annotation of tumor (blue) and stroma (orange) regions in two ROIs with the highest deviation between m.e. observers (average of seniors 2 and 3) and AI-based TSR values. Both ROIs belong to category A in Figure 6. From left to right: original ROI ID 19, ROI ID 19 with annotation overlay, original ROI ID 16, original ROI ID 16 with annotation overlay.

**Figure A3:**
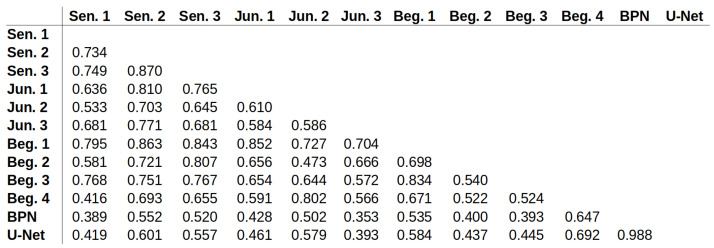
ICC values for all possible pairs, including both observers and AI.

**Figure A4:**
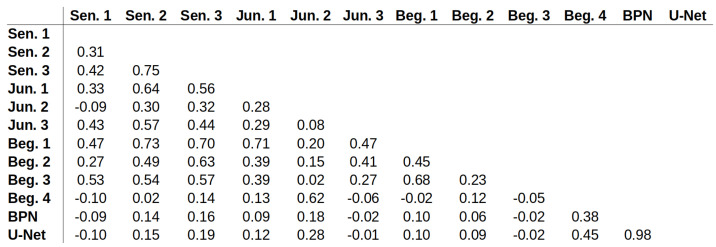
Lower value of 95% CI of ICC values for all possible pairs, including both observers and AI.

**Figure A5:**
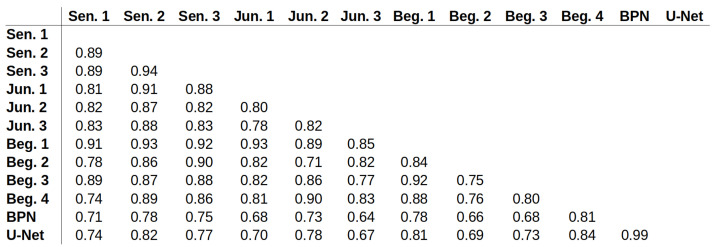
Upper value of 95% CI of ICC values for all possible pairs, including both observers and AI.

In order to assess the concordance of the AI-TSR and the observers’ TSR to compare it to the concordance between observers, all pair-wise ICC values were calculated and are given in the Appendix (Figure A3, Figure A4 and Figure A5) including the lower and upper bounds of the 95% CI interval. The pairwise ICC between the BPN model and “senior 2” was 0.552 (95% CI 0.14–0.78) and that between the BPN model and “senior 3” was 0.520 (95% CI 0.16–0.75), showing moderate agreement, with a significant uncertainty witnessed by large confidence intervals. “Senior 2” and “senior 3” are the two most experienced observers. The confidence interval for the pair-wise ICC values between two observers, or between observers and AIs, was high for most pairs, which is partly due to the fact that it is designed to measure the agreement within a group of more than two observers. Nevertheless, the ICC values between the observer and AI (BPN and U-Net) were lower than those between individual observers (see Figure A3, Figure A4 and Figure A5). The reduced concordance between the observer and AI was mainly caused by two ROIs with large deviations in TSR values between the m.e. observers and AI of more than 50 percentage points (ROI ID 16 and 19 in Figure 4). Therefore, these ROIs were additionally annotated manually (Figure 5) and are discussed in detail later on.

Calculating the ICC without these two ROIs led to ICCs between the BPN model and the two m.e. observers of 0.750 (95% CI 0.33–0.90) and 0.689 (95% CI 0.38–0.85), respectively. The Cohen’s kappa values (applying a cutoff of 50% to generate the categorized TSR) between the BPN model and the two m.e. observers were 0.400 and 0.333, respectively, showing only fair to moderate agreement. Using a cutoff of 65% yielded kappa values of 0.502 for both observers, showing moderate agreement. For a cutoff of 50%, our Cohen’s kappa values are higher than those of Geessink et al. (2019) [8] with 0.239, but they are lower than their value of 0.521 with a threshold of roughly 65% and 0.623 from Hong et al. [28].

**Figure 6:**
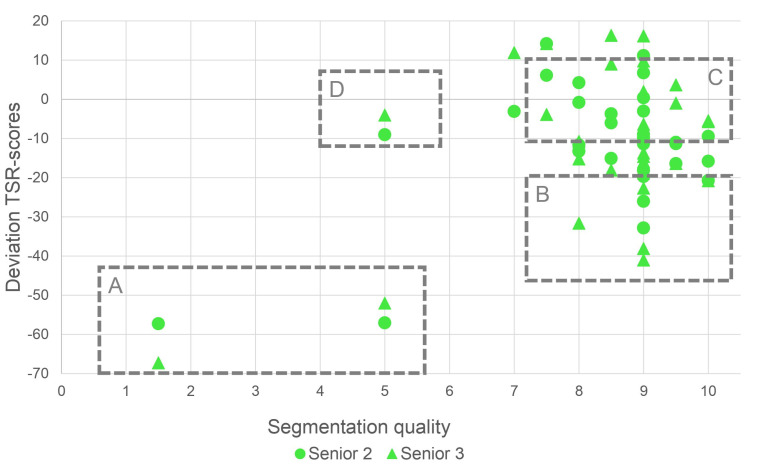
The differences between m.e. observers and automated TSR estimations (BPN) are plotted against the rated segmentation quality for each ROI. There are roughly 4 categories into which the results can be classified. Category A: poor segmentation quality rating and very high deviation between AI-TSR and estimated TSR. Category B: good segmentation quality rating but high deviation between AI-TSR and estimated TSR. Category C: good segmentation quality rating and minor deviation between AI-TSR and estimated TSR. Category D: poor segmentation quality rating but minor deviation between AI-TSR and estimated TSR.

In order to assess whether differences between observer TSR values and AI-TSR values were due to inaccuracies of human estimation or incorrect segmentation results of the AI-models, the results of the second survey were used. In this survey, two observers rated the segmentation quality obtained using the BPN approach for all 30 ROIs. The assumption was that the evaluation of the segmentation result might be conducted more reliably than the area estimation of tumor and stroma components in an ROI. Figure 6 shows the deviation between AI-TSR values (BPN approach) and each of the two most experienced observers plotted against the rated segmentation quality. Four categories (A to D) can be distinguished. Category A comprises ROIs that show a low rating of segmentation quality and a significant deviation between the AI-TSR and m.e. observers TSR. The poor segmentation rating suggested that the AI-TSR was incorrect, and one would lean toward the observer assessment. However, further investigations carried out later showed that neither was correct in their assessment for ROIs of category A. Category B comprises ROIs with a high rating of segmentation quality but a significant deviation to the m.e. observers TSR of more than 20 percentage points. Considering the good segmentation ratings, the AI-TSR was likely correct (or at least close), which in turn implies the observer assessment was biased. All ROIs in category C have a small deviation between AI-TSR and m.e. observers TSR values, and the segmentation quality rating is high enough to assume the AI-TSR as well as the human estimations were correct. Finally, the ROI contained in category D is characterized by a poor segmentation quality rating with only small deviation in TSR values indicating that both human and AI-TSR values were error-prone.

Figure 4b and Figure 6 show that the AI-TSR tends to be lower than the TSR estimation of the m.e. observers (or all observers). Without the information provided by the second survey, this might suggest a bias in the AI-based segmentation approach. Considering only the 17 ROIs for which the segmentation quality was rated 9.0 or above by human observers, the mean deviation between the TSR obtained by the BPN model and the m.e. observers is −11.5 (“senior 2”) and −11.1 (“senior 3”) percentage points, respectively.

This perspective suggests a bias in human estimations, specifically an overestimation of the TSR. A possible explanation is that the task of estimation of a TSR can be divided into two subtasks: (1) recognizing tissue types and (2) estimating their ratio. Whereas survey 1 (“Comparison between Human Estimation and Automated TSR assessment”) includes both subtasks, survey 2 (“Assessment of segmentation quality”) yields insights on subtask 1 alone. Cases where the segmentation quality was judged to be good, but the resulting TSR deviate significantly, indicate that subtask 2—estimation of area ratios—was prone to human error.

**Figure 7:**
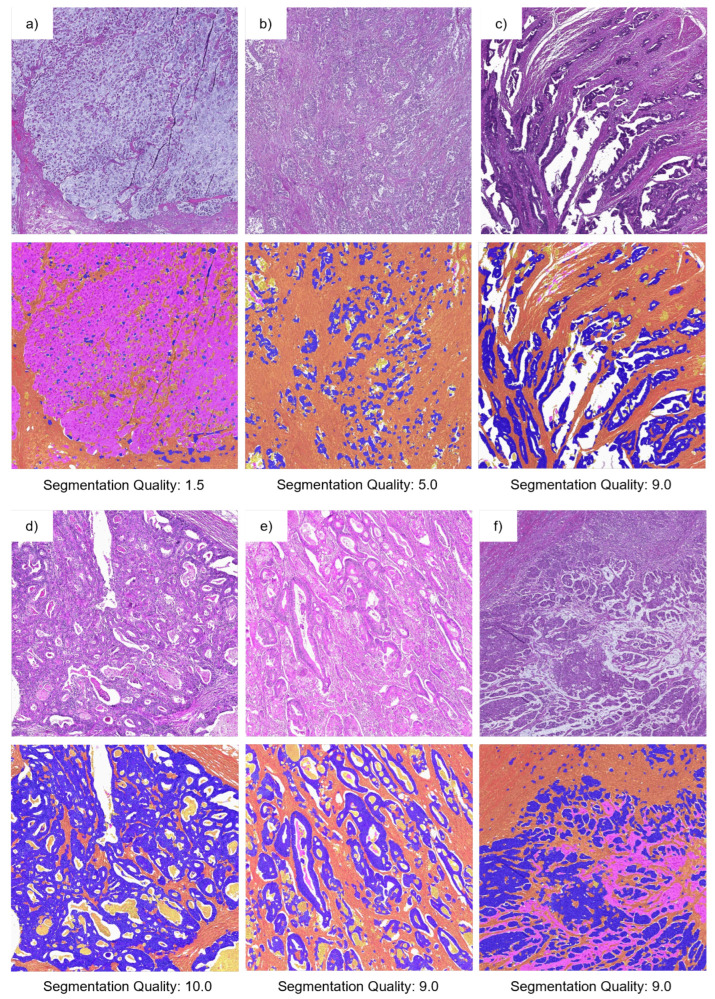
Original ROIs (first and third row) and the corresponding segmentation (BPN approach) results (second and fourth row) as color-coded overlay. Sub-figures (**a**–**f**) show different ROIs. Each ROI has a size of 2 mm × 2 mm. Class labels are color-coded: blue (tumor), orange (stroma), yellow (necrosis), magenta (mucus), white (background). The mean segmentation quality assessed by two observers for these ROIs is given below the corresponding segmentation results. The rating ranged from 1 (insufficient) to 10 (excellent).

Examples of segmentation results with different ratings and the corresponding original ROIs are shown in Figure 7. The first example (a) had a poor segmentation quality of only 1.5, with a deviation in TSR of −57.3 and −67.3 percentage points to the m.e. observers, respectively, and it shows a special subtype of colon cancer: a signet ring cell carcinoma. The stroma, mostly on the bottom and left side of the image, is easily recognizable. However, the BPN approach misinterpreted most signet ring cells as mucus. The massive deviation in TSR estimation hence is to be expected, given that signet ring cells are present throughout the mucus. The most likely reason for the missegmentation observed is that the signet ring cell subtype, especially with tumor cells surrounded by mucus, was not present in the training data. Therefore, the model had no way of learning the characteristics necessary for an accurate segmentation. The second ROI (b) had a segmentation quality rating of 5.0 and a deviation in TSR of −57.0 and −52.0 percentage points, respectively. The BPN approach detected only part of the tumor, misclassifying part of it as stroma. Both examples are contained in category A of Figure 6. The deviations in TSR values between AI and m.e. observers for the ROI in (c) (6.8 and 9.8 percentage points, respectively) and for the ROI in (d) (−9.4 and −5.4 percentage points, respectively) were small while having high segmentation quality (rated with 9.0 and 10.0, respectively). Both ROIs (c) and (d) are examples of category C. The two ROIs in (e) and (f) show examples of category B. The ROI in (e) had a segmentation quality rating of 9.0 but a deviation of −26.0 and −41.0 percentage points, respectively. The last ROI (f) also had a rated segmentation quality of 9.0 with a deviation of −32.8 and −18.8 percentage points, respectively. Especially the ROIs in (d) and (e) show that with our approach, TSR values can be determined even for areas containing necrosis and mucus.

Finally, we had a closer look at the two ROIs with the highest deviation between the m.e. observers TSR and the AI-TSR values. Both ROIs (ID 16 and ID 19) belong to category A in Figure 6. Within these two ROIs, stroma and tumor tissue was manually annotated (Figure 5), and a TSR value was calculated based on these manual annotations. In Table 4, the various TSR values for these two ROIs are given. Assuming that the TSR value derived from the manual annotations is the gold standard, TSR values obtained by both human observers and the AI were compared to it. The respective deviations are given in Table 5. The comparison showed that the TSR estimates of the human observers as well as the AI-TSR showed considerable deviations from the TSR value based on the manual annotations.

## 4. Conclusions

In this work, we presented two AI-based approaches (BPN and U-Net) to segment ROIs from H&E-stained colon sections into five classes (tumor, stroma, necrosis, mucus and background). Both approaches achieved a high accuracy of 86.5% and 86.7%, respectively, when evaluated pixel-wise on a test dataset. The tumor and stroma classes were particularly well segmented (with F${}_{1}$ scores of 0.921 and 0.895 (BPN) and 0.923 and 0.894 (U-Net)), whereas segmentation of the other classes showed some limitations. Although the U-Net approach performed slightly better than the BPN approach, the BPN approach offers the advantage of adaptability to new tasks. The capability of these approaches to determine a reliable TSR score was investigated in detail on a second dataset consisting of 30 ROIs with a size of 2 mm × 2 mm from 30 different WSIs.

In direct comparison to ten human observers, the AI-TSR values achieved a lower agreement (ICC as well as Cohen’s kappa) with the TSR values of the most experienced observers. However, a further investigation showed that a good agreement of the TSR values between the human observers does not necessarily mean that these TSR values also objectively reflect the area ratios of tumor and stroma fractions in the ROI under consideration. An assessment of the quality of the segmentation results by two human observers (ranging from 1 (insufficient) to 10 (excellent)) yielded a rating of at least 7 for 90% of the ROIs, and for 57%, it yielded even a rating of at least 9.

In conclusion, a major challenge in the development of automated TSR scoring approaches is the generation of a reliable ground truth for the validation of these approaches. The creation of a pixel-precise ground truth is very time consuming, but TSR scores estimated by human observers are subject to bias. In this context, the addition of a qualitative assessment of the segmentation quality can be useful. Overall, the evaluation results are promising, especially if the training data are further extended to include additional subtypes such as signet ring cells.

Further future work will focus on (i) overcoming the limitation that the proposed BPN model predicts the segmentation mask in a limited resolution, (ii) the evaluation of the few-shot model’s adaptability to other segmentation tasks, and (iii) aiming toward an autonomous system that does not require human interaction.

## Figures and Tables

**Table 1:** Distribution of different classes across the three datasets. The number of pixels assigned to each class is stated. The artifact class was introduced for regions that should be ignored during the training and evaluation.

Dataset	# Tumor	# Stroma	# Necrosis	# Mucus	# Background	# Artifact
training	306,215,439	226,924,661	36,776,447	33,136,244	77,120,642	3,289,067
validation	67,367,966	47,092,974	11,588,088	10,367,879	29,945,593	0
test	86,969,626	71,515,442	10,713,472	9,694,276	20,507,721	3,691,963

**Table 2:** Results of pixel-wise evaluation on segmentation test set. All classes were weighted equally in the averaging, regardless of how many pixels belong to that class.

Metric	BPN (Tiling A)	U-Net (Tiling A)	BPN (Tiling B)	U-Net (Tiling B)
accuracy	0.856 ± 0.007	0.858 ± 0.004	0.865 ± 0.005	**0.867** ± **0.004**
precision	0.784 ± 0.009	0.785 ± 0.007	**0.801** ± **0.003**	0.795 ± 0.007
recall	0.748 ± 0.014	0.755 ± 0.003	0.751 ± 0.021	**0.767** ± **0.003**
IoU	0.629 ± 0.007	0.635 ± 0.004	0.637 ± 0.017	**0.650** ± **0.003**
F${}_{1}$ score	0.761 ± 0.011	0.766 ± 0.003	0.765 ± 0.016	**0.777** ± **0.003**

**Table 3:** F${}_{1}$ score for individual classes and for pixel-wise segmentation.

Class	BPN (Tiling A)	U-Net (Tiling A)	BPN (Tiling B)	U-Net (Tiling B)
tumor	0.913 ± 0.006	0.916 ± 0.004	0.921 ± 0.004	**0.923** ± **0.004**
stroma	0.881 ± 0.012	0.883 ± 0.007	**0.895** ± **0.007**	0.894 ± 0.006
necrosis	0.634 ± 0.020	0.622 ± 0.008	**0.655** ± **0.013**	0.638 ± 0.009
mucus	0.663 ± 0.061	0.695 ± 0.020	0.638 ± 0.069	**0.712** ± **0.019**
background	0.715 ± 0.004	0.715 ± 0.004	0.718 ± 0.004	**0.719** ± **0.005**
overall	0.761 ± 0.011	0.766 ± 0.003	0.765 ± 0.016	**0.777** ± **0.003**

**Table 4:** Comparison of TSR values for two ROIs with the highest deviation between m.e. observer TSR (averaged over the two m.e. observers) and AI-based TSR. Additionally, the TSR (“Manual”) was calculated based on manual annotation of tumor and stroma.

ROI ID	Manual	m.e. Observers	BPN	U-Net
16	47.6%	72.5%	18.0%	21.1%
19	41.8%	75.0%	12.7%	24.8%

**Table 5:** Deviation of different TSR values to the “manual” TSR value derived from the manual annotations for two selected ROIs. The deviation is noted in percentage points.

ROI ID	m.e. Observers	BPN	U-Net
16	24.9 pp	−29.6 pp	−26.5 pp
19	33.2 pp	−29.1 pp	−17.0 pp

**Figure A2:**
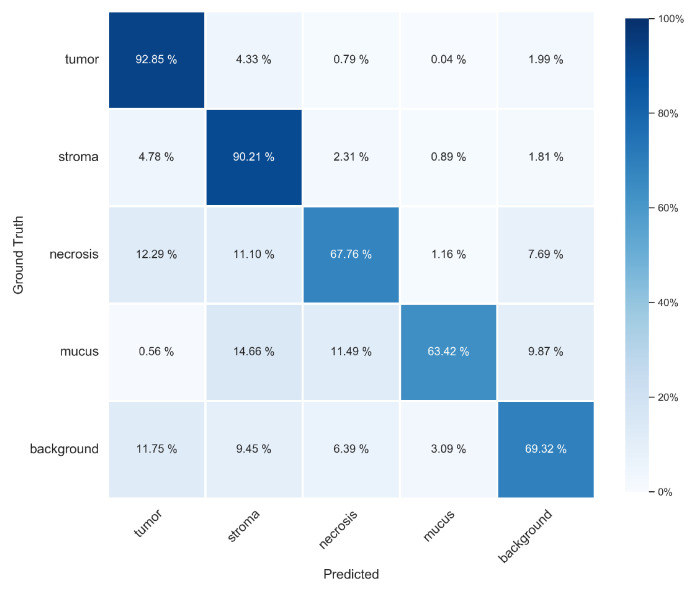
Confusion matrix obtained with the U-Net model on the segmentation test set applying tiling B. The rows represent the ground truth class labels, and the columns represent the predictions. Due to high imbalances in the number of pixels per class, a relative representation of the confusion matrix was chosen. Thereby, the rows of the matrix are each normalized to 100% based on the number of pixels of each class in the test set.

## Data Availability

The image data of the survey together with the estimated and calculated TSR values are available on reasonable request by contacting the corresponding authors.

## Abbreviations

The following abbreviations are used in this manuscript:

AI	artifical intelligence
BPN	basic prototype network
CI	confidence interval
CNN	convolutional neural network
DAB	diaminobenzidine
GAN	generative adversarial network
H&E	hematoxylin and eosin
ICC	intraclass correlation
IoU	intersection over union
m.e.	most experienced
PANet	prototype alignment network
ROI	region of interest
SVM	support vector machine
TMA	tissue microarray
TME	tumor microenvironment
TSR	tumor–stroma ratio
WSI	whole slide image

## Appendix A. Additional Information for U-Net Structure

The number of filters for all convolutional layers in the expansive path is 48, 48, 32, 32, 24, 24, 16, 16, and 5.

## Appendix B. Additional Information about the Surveys

The surveys were conducted online using LimeSurvey. All ROIs were available in native resolution. An example view is depicted in Figure A1.

## Appendix C. Evaluation Metrics

The pixel-wise comparison of the ground truth labels with the corresponding predicted labels results in a confusion matrix. According to the five classes that are considered, it has five columns and five rows (Figure 2). To calculate evaluation metrics such as precision or recall for a specific class, a two-class confusion matrix (Table A1) has to be derived from the original five-class confusion matrix. For instance, if the metrics are to be calculated for the tumor class, this class will be considered as the positive class, and all four other classes will be summarized in the negative class. Afterwards, precision, recall and F${}_{1}$ score are calculated as defined in Equations (A1)–(A3).

**Table A1:** Confusion matrix for a two-class setting with one positive class (P) and a negative class (N). “True positives” (TP) are all instances of class P that are correctly predicted. “False negatives” (FN) are all instances of class P that are incorrectly predicted to belong to class N. “False positives” are all instances of class N that are incorrectly predicted to belong to class P. “True negatives” denotes all instances of class N that are correctly predicted.

		Predicted	
		**P**	**N**
**Actual**	**P**	TP	FN
	**N**	FP	TN

(A1)
$$\mathrm{precision}=\frac{\mathrm{TP}}{\mathrm{TP}+\mathrm{FP}}$$

(A2)
$$\mathrm{recall}=\frac{\mathrm{TP}}{\mathrm{TP}+\mathrm{FN}}$$

(A3)
$$F{}_{1}\;\mathrm{score}=\frac{2*\mathrm{precision}*\mathrm{recall}}{\mathrm{precision}+\mathrm{recall}}=\frac{2\mathrm{TP}}{2\mathrm{TP}+\mathrm{FP}+\mathrm{FN}}$$

## Appendix D. Additional Results of Pixel-Wise Evaluation on Segmentation Test Set

**Table A2:** Recall for individual classes for pixel-wise evaluation on segmentation test set.

Class	BPN (Tiling A)	U-Net (Tiling A)	BPN (Tiling B)	U-Net (Tiling B)
tumor	0.914 ± 0.016	0.918 ± 0.011	0.925 ± 0.014	**0.929** ± **0.009**
stroma	0.897 ± 0.006	0.897 ± 0.005	**0.910** ± **0.009**	0.902 ± 0.004
necrosis	0.648 ± 0.034	0.655 ± 0.062	**0.682** ± **0.027**	0.678 ± 0.062
mucus	0.582 ± 0.100	0.618 ± 0.033	0.525 ± 0.115	**0.634** ± **0.029**
background	0.701 ± 0.014	0.689 ± 0.028	**0.710** ± **0.012**	0.693 ± 0.028
overall	0.748 ± 0.014	0.755 ± 0.003	0.751 ± 0.021	**0.767** ± **0.003**

**Table A3:** Precision for individual classes for pixel-wise evaluation on segmentation test set.

Class	BPN (Tiling A)	U-Net (Tiling A)	BPN (Tiling B)	U-Net (Tiling B)
tumor	0.912 ± 0.006	0.914 ± 0.004	0.917 ± 0.006	**0.918** ± **0.003**
stroma	0.866 ± 0.021	0.870 ± 0.010	0.881 ± 0.020	**0.886** ± **0.008**
necrosis	0.620 ± 0.017	0.601 ± 0.046	**0.631** ± **0.018**	0.611 ± 0.044
mucus	0.792 ± 0.033	0.797 ± 0.010	**0.848** ± **0.046**	0.811 ± 0.011
background	0.731 ± 0.015	0.746 ± 0.026	0.727 ± 0.017	**0.749** ± **0.025**
overall	0.784 ± 0.009	0.785 ± 0.007	**0.801** ± **0.003**	0.795 ± 0.007

**Table A4:** Intersection over union for individual classes for pixel-wise evaluation on segmentation test set.

Class	BPN (Tiling A)	U-Net (Tiling A)	BPN (Tiling B)	U-Net (Tiling B)
tumor	0.788 ± 0.019	0.791 ± 0.010	**0.810** ± **0.012**	0.808 ± 0.010
stroma	0.840 ± 0.010	0.845 ± 0.007	0.854 ± 0.007	**0.858** ± **0.006**
necrosis	0.464 ± 0.021	0.452 ± 0.009	**0.487** ± **0.014**	0.469 ± 0.009
mucus	0.499 ± 0.066	0.533 ± 0.023	0.472 ± 0.076	**0.553** ± **0.022**
background	0.557 ± 0.005	0.557 ± 0.005	0.560 ± 0.005	**0.562** ± **0.006**
overall	0.629 ± 0.007	0.635 ± 0.004	0.637 ± 0.017	**0.650** ± **0.003**
